# High life in the sky? Mortality by floor of residence in Switzerland

**DOI:** 10.1007/s10654-013-9809-8

**Published:** 2013-05-10

**Authors:** Radoslaw Panczak, Bruna Galobardes, Adrian Spoerri, Marcel Zwahlen, Matthias Egger

**Affiliations:** 1 Institute of Social and Preventive Medicine (ISPM), University of Bern, Finkenhubelweg 11, 3012 Bern, Switzerland; 2 School of Social and Community Medicine, University of Bristol, Bristol, UK

**Keywords:** Floor of residence, Housing, High-rise building, Mortality, Suicide, Cohort study, Switzerland

## Abstract

Living in high-rise buildings could influence the health of residents. Previous studies focused on structural features of high-rise buildings or characteristics of their neighbourhoods, ignoring differences within buildings in socio-economic position or health outcomes. We examined mortality by floor of residence in the Swiss National Cohort, a longitudinal study based on the linkage of December 2000 census with mortality and emigration records 2001–2008. Analyses were based on 1.5 million people living in buildings with four or more floors and 142,390 deaths recorded during 11.4 million person-years of follow-up. Cox models were adjusted for age, sex, civil status, nationality, language, religion, education, professional status, type of household and crowding. The rent per m^2^ increased with higher floors and the number of persons per room decreased. Mortality rates decreased with increasing floors: hazard ratios comparing the ground floor with the eighth floor and above were 1.22 [95 % confidence interval (CI) 1.15–1.28] for all causes, 1.40 (95 % CI 1.11–1.77) for respiratory diseases, 1.35 (95 % CI 1.22–1.49) for cardiovascular diseases and 1.22 (95 % CI 0.99–1.50) for lung cancer, but 0.41 (95 % CI 0.17–0.98) for suicide by jumping from a high place. There was no association with suicide by any means (hazard ratio 0.81; 95 % CI 0.57–1.15). We conclude that in Switzerland all-cause and cause-specific mortality varies across floors of residence among people living in high-rise buildings. Gradients in mortality suggest that floor of residence captures residual socioeconomic stratification and is likely to be mediated by behavioural (e.g. physical activity), and environmental exposures, and access to a method of suicide.

**Electronic supplementary material:**

The online version of this article (doi:10.1007/s10654-013-9809-8) contains supplementary material, which is available to authorized users.

## Introduction

Housing is an important determinant of health, which has attracted considerable interest in public health research and policy. The World Health Organization (WHO) has identified inadequate housing conditions as an important factor contributing to injuries and preventable diseases such as cancer, respiratory, nervous system and cardiovascular diseases [1]. Housing conditions may affect health both directly and indirectly. Direct effects on health might operate through the biological, chemical or physical characteristics of buildings such as the presence of radon, asbestos or pests, unsafe heating systems, overcrowding or indoor pollution [2, 3]. Indirect effects might act through individual characteristics and exposures related to the socio-economic position of those living in a building and neighbourhood [4, 5]. Indeed, housing tenure, amenities and conditions are widely used as a proxy for socio-economic position at the individual or family level [6–9].

High-rise housing is of particular concern to public health. An estimated one in six of European dwellings (some 36 millions) is located in high-rise buildings [10]. Most of these buildings originate from the high-rise construction boom of the 1960s and 1970s and many are in poor condition, located in more deprived areas and include a significant share of social housing [11]. The influence of high-rise housing on the health of individuals and communities has been a matter of debate for decades. For example, in the 1970s some architects claimed that “there is abundant evidence to show that high buildings make people crazy” [12, p. 115]. The reputation of high-rise housing as unpleasant and unhealthy habitats that isolate people from their social environment and increase crime continues to the present. In Switzerland and other industrialized countries, the construction of high-rise buildings has experienced a revival in recent years in the context of dwindling land resources in urban centres. Unlike the tower blocks of the 1960s and 1970s, these are often glitzy buildings located in prime locations that include offices and shops in the lower floors and luxury apartments on the upper and top floors. In Switzerland and elsewhere living higher up in a high-rise building is more prestigious than living in one of the lower floors, with rents increasing with floors and the most desirable flats located on the top floor.

Most previous studies of high-rise housing and health focused on structural features of high-rise buildings or characteristics of their neighbourhoods, largely ignoring differences within buildings in socio-economic position or health outcomes. Even fewer studies reported effects of floor of residence on health outcomes [13]. We used data from the Swiss National Cohort [14, 15] to examine the association of the floor of residence on all-cause and cause specific mortality in Switzerland.

## Methods

### The Swiss National Cohort

Described in detail elsewhere [14, 15], the Swiss National Cohort (SNC) is a national longitudinal study of mortality based on the linkage of census data with mortality and emigration records. Participation in the census is compulsory in Switzerland and for the 2000 census coverage was estimated to be 98.6 % [16]. In the absence of names or a unique personal identifier, linkage used a combination of deterministic and probabilistic methods based on sex, date of birth, marital status, nationality, religion, place of residence and other variables [14, 15].

The database analysed for this study consisted of census 2000 data that were linked to deaths and emigration records up to the end of 2008. The census consists of three questionnaires: one for the individual person, a household questionnaire and a questionnaire on the building. All questionnaires and a complete list of variables are available at http://www.swissnationalcohort.ch. The floor of each dwelling was recorded on the household questionnaire. The building questionnaire provided information on the total number of floors in the building. In order to examine the gradient of mortality across floors we restricted our analyses to residents of buildings with at least four floors. We included persons aged 30–94 years who participated in the census of 5th December 2000. We excluded persons aged below 30 because linkage is less complete in this age group [14] and some individuals may still be in (tertiary) education. We also excluded individuals living in institutions, individuals with no exact information on the floor of residence, people living in temporary or provisional housing and people with missing information on the highest achieved education.

### Variables

We grouped civil status into categories ‘Single’, ‘Married’, ‘Widowed’ and ‘Divorced’. Nationality was in three categories: ‘Swiss’, ‘Europe other than Switzerland’ and ‘Other/unknown’; religion in four: ‘Protestant’, ‘Roman Catholic’, ‘No religious affiliation’ and ‘Other/unknown’ and spoken language also in four: ‘German’, ‘French’, ‘Italian’ and ‘Other’. We grouped highest educational achievement as ‘Primary or less’, ‘Secondary’ or ‘Tertiary’. We collapsed the 33 grade socio-professional categorisation of occupations developed by the Swiss Federal Statistical Office [17] into eight categories of socio-professional status capturing skill level and position of individuals on the labour market. Type of household was divided into four categories on the basis of the number of adults and children in the household: ‘Single person household’, ‘Couple without children’, ‘Household with one or more children’, ‘Other’. We grouped household ownership type into ‘Rented flat’, ‘Owned flat’ and ‘Other’. Household crowding was defined as the total number of persons per number of rooms and treated as a continuous variable. The floors in the buildings were categorized into nine levels: ‘Ground floor’, floors 1–7 and floor 8 and above. Flats located on the ground floor, raised ground floor and basement were combined in the category ‘Ground floor’.

### Mortality

We explored associations of floor of residence at the time of census with all-cause and cause specific mortality between 5th December 2000 and 31st December 2008. The deaths were coded according to the tenth revision of the International Classification of Deaths, Injuries and Causes of Death (ICD-10). Outcomes were deaths from all causes, cardiovascular diseases (ICD-10 codes I00-I99), myocardial infarction (ICD-10 codes I21-I22), stroke (ICD-10 codes I60-I64), respiratory diseases (ICD-10 codes J00-J99), alcohol related deaths (ICD-10 codes F10, G31.2, G62.1, I42.6, K29.2, K70, K73, K74 (excluding K74.3-K74.5, K86.0, X45, X65, Y15) [18], stomach cancer (ICD-10 code C16), lung cancer (ICD-10 codes C33–C34), breast cancer (ICD-10 code C50), prostate cancer (ICD-10 code C61), transport accidents (ICD-10 codes V01–V99), suicide (ICD-10 codes X60–X84) and suicide by jumping from a high place (ICD-10 code X80).

### Ethics

The SNC was approved by the cantonal ethics committees of Bern and Zurich, with the approval covering the present analysis.

### Statistical analysis

We modelled the hazard ratio of death across floors of residence for all-cause mortality and specific causes of death using Cox regression models. Time of observation was from the date of census (5th December 2000) to the date of death, the date of emigration or 31st December 2008, whichever came first. We adjusted for age by using age as the time scale in the models. We compared the residents of ground floors to those living on the eighth floor or higher. We used two models with different levels of adjustments: (1) adjusted for age and sex, (2) adjusted for age, sex, civil status, nationality, language, religion, education, professional status, type and ownership of household and crowding (fully adjusted). Models were stratified by building, thus allowing the baseline hazard to differ between different buildings. Stratification by building also meant that analyses were controlled for degree of urbanicity, language region, and socio-economic position of the neighbourhood.

In additional analyses we examined whether the association between mortality and floor of residence was modified by the socio-economic standing of the area. We used the Swiss neighbourhood index of socioeconomic position (Swiss-SEP) [19] for this purpose. Swiss-SEP is a composite measure based on four domains (income, education, occupation and housing) which describes the socio-economic position of 1.27 million overlapping neighbourhoods. We calculated a log- likelihood ratio test of interaction for this purpose, entering quintiles of the Swiss-SEP index. We also explored whether the observed associations might be due to reverse causality, where sicker individuals choose to live on lower floors whereas healthier people tend to live on higher floors. Firstly, we narrowed the study population to individuals who had lived on the same floor for 5 years or longer prior to the census, thus excluding those who moved to the high rise more recently. Secondly, we assessed whether the association of floor of residence with all-cause mortality differed between the first 4 years of follow up (5th of December 2000 and 5th of December 2004) and the second 4 years (6th of December 2004–31st December 2008). The results are reported as hazard ratios (HR) with 95 % confidence intervals (CI). All statistical analyses were done in Stata version 12 (Stata Corporation, College Station, TX, USA).

## Results

**Fig. 1:**
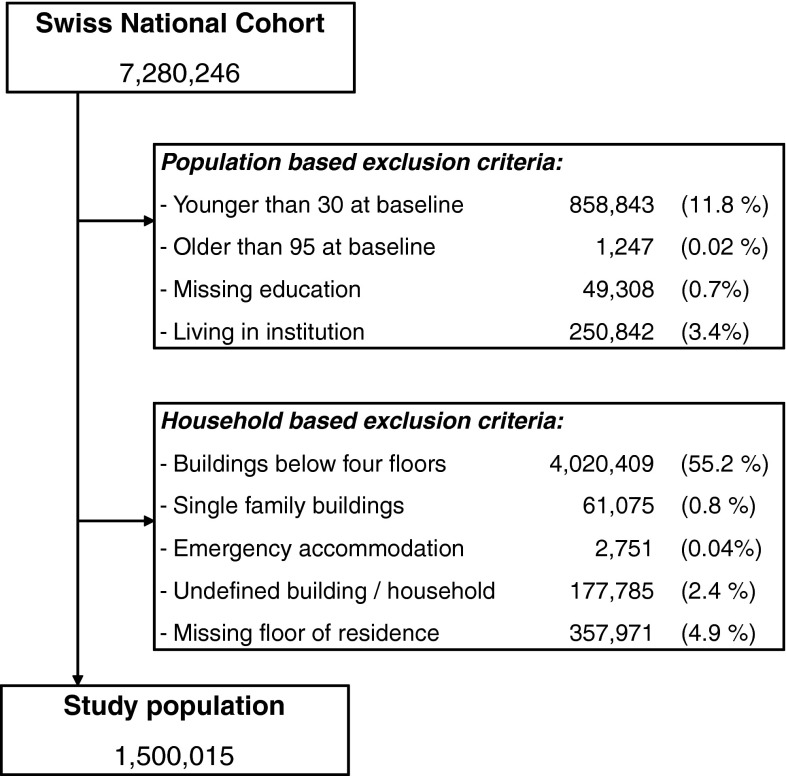
Flowchart of selecting the eligible study population

The SNC includes 7,280,246 people who participated in the 2000 census. For the present analysis we excluded 858,843 (11.8 %) persons younger than 30 years and 1,247 (0.02 %) individuals older than 95 years at the time of census. We also excluded 4,081,484 (56.1 %) residents of buildings with fewer than four floors and 357,971 (4.9 %) individuals with missing information on the floor of residence. Furthermore, we excluded 49,308 (0.7 %) persons with missing information on education, 250,842 (3.4 %) individuals living in institutions, 177,785 (2.4 %) people with incomplete information on their household and 2,751 (0.04 %) people living in temporary or provisional housing. Our study population thus consisted of 1,500,015 persons living in 1,008,190 households and 160,629 buildings with four or more floors. The identification of eligible individuals is summarized in Fig. 1.

Women, older people and divorced and widowed individuals were more likely to be residents of higher floors than younger individuals and married people (Table 1). These differences were also reflected in the composition of households: there were more single person households and households consisting of couples without children on higher floors compared to lower floors. Furthermore, the percentage of Swiss nationals, French speaking residents and persons with no religious affiliation increased with the number of floors. There were few differences in the distribution of educational level or professional status across floors, however, the percentage of persons not in paid employment increased from 31.6 % on the ground floor to 46.3 % on the eighth floor and above.

**Table 1:** Characteristics of the study population across first fourth and eight and higher floor of residence

Characteristic	Ground floor		Fourth floor		Eighth floor		Total	
	N	%	N	%	N	%	N	%
Total	218,529	100.0	158,605	100.0	42,246	100.0	1,500,015	100.0
Gender								
Male	105,694	48.4	74,308	46.9	19,257	45.6	703,972	46.9
Female	112,835	51.6	84,297	53.1	22,989	54.4	796,043	53.1
Age (years)								
Mean (SD)	50.4	(14.9)	53.0	(15.5)	56.7	(15.2)	52.3	(15.4)
30–39	68,098	31.2	40,843	25.8	7,340	17.4	410,355	27.4
40–49	53,303	24.4	34,188	21.6	7,585	18.0	334,038	22.3
50–64	56,114	25.7	44,950	28.3	13,821	32.7	411,497	27.4
65–94	41,014	18.8	38,624	24.4	13,500	32.0	344,125	22.9
Civil status								
Single	35,041	16.0	31,347	19.8	6,255	14.8	262,071	17.5
Married	143,024	65.4	94,795	59.8	26,212	62.0	940,662	62.7
Widowed	16,127	7.4	13,719	8.6	4,453	10.5	127,488	8.5
Divorced	24,337	11.1	18,744	11.8	5,326	12.6	169,794	11.3
Nationality								
Switzerland	158,602	72.6	117,958	74.4	32,828	77.7	1,104,671	73.6
Rest of Europe	53,734	24.6	35,732	22.5	8,057	19.1	351,856	23.5
Other/unknown	6,193	2.8	4,915	3.1	1,361	3.2	43,488	2.9
Religion								
Protestant	70,769	32.4	45,956	29.0	14,277	33.8	460,813	30.7
Catholic	93,286	42.7	69,418	43.8	16,021	37.9	653,289	43.6
No affiliation	29,466	13.5	25,384	16.0	7,069	16.7	213,598	14.2
Other/unknown	25,008	11.4	17,847	11.3	4,879	11.5	172,315	11.5
Language								
German	124,531	57.0	81,358	51.3	20,756	49.1	813,135	54.2
French	46,436	21.2	44,120	27.8	14,732	34.9	371,097	24.7
Italian	19,193	8.8	13,910	8.8	1,879	4.4	126,893	8.5
Other	28,369	13.0	19,217	12.1	4,879	11.5	188,890	12.6
Education								
Primary or less	62,214	28.5	42,926	27.1	11,747	27.8	424,767	28.3
Secondary	110,182	50.4	78,688	49.6	22,183	52.5	750,850	50.1
Tertiary	46,133	21.1	36,991	23.3	8,316	19.7	324,398	21.6
Professional status								
Top management, independent professions	4,820	2.2	3,524	2.2	645	1.5	31,453	2.1
Other self-employed	11,728	5.4	7,866	5.0	1,371	3.2	71,920	4.8
Professionals, senior management	12,096	5.5	10,351	6.5	1,897	4.5	86,855	5.8
Lower management, skilled labour	61,493	28.1	39,401	24.8	9,477	22.4	391,552	26.1
Unskilled labour	20,299	9.3	12,629	8.0	3,157	7.5	129,790	8.7
Other employment	32,434	14.8	21,217	13.4	4,955	11.7	205,891	13.7
Unemployed, seeking work	6,707	3.1	5,068	3.2	1,178	2.8	45,819	3.1
Not in paid employment	68,952	31.6	58,549	36.9	19,566	46.3	536,735	35.8
Type of household								
Single person household	54,994	25.2	48,704	30.7	12,651	29.9	416,550	27.8
Couple without children	65,190	29.8	53,881	34.0	16,477	39.0	496,043	33.1
Household with children	82,648	37.8	44,469	28.0	10,398	24.6	479,328	32
Single parent household	10,647	4.9	6,716	4.2	1,698	4.0	68,520	4.6
Other	5,050	2.3	4,835	3.0	1,022	2.4	39,574	2.6

Swiss National Cohort, Switzerland 2000

As expected, the rent (in CHF per m^2^) increased with the number of floors (Table 2). The size of flats decreased with increasing number of floors but the average number of persons per room was nevertheless lower at higher floors: it decreased from 0.72 person per room on the ground floor to 0.63 person per room on the eighth floor and above. Of note, almost half (44.7 %) of our study population lived in buildings with four floors compared to 14.0 % living in buildings with eight or more floors (Webtable 1). The majority of the buildings included in our study were located in urban (46.8 %) and peri-urban (35.4 %) communities.

**Table 2:** Characteristics of households and flats across floors of residence in high-rise buildings

Floor	No. of households		Size of flats (m^2^)^a^		Crowding (persons/room)		Rent (CHF/m^2^)^a^	
	N	%	Mean	SD	Mean	SD	Mean	SD
Ground floor	144,839	14.4	83	(37)	0.72	(0.43)	13.65	(4.99)
Floor 1	215,727	21.4	82	(32)	0.68	(0.40)	13.66	(4.92)
2	222,057	22.0	83	(32)	0.66	(0.38)	13.79	(5.01)
3	190,437	18.9	82	(33)	0.66	(0.38)	13.96	(5.12)
4	108,493	10.8	81	(34)	0.66	(0.40)	14.32	(5.35)
5	53,230	5.3	79	(33)	0.67	(0.40)	14.64	(5.24)
6	28,500	2.8	79	(33)	0.67	(0.41)	14.44	(4.86)
7	16,494	1.6	79	(31)	0.65	(0.39)	14.47	(4.77)
8+	28,413	2.8	78	(30)	0.63	(0.37)	14.14	(4.26)
Total	1,008,190	100.0	82	(33)	0.67	(0.40)	13.92	(5.04)

Swiss National Cohort, Switzerland 2000

^a^Information on rent and size of flats was available for 681,902 (67.6 %) flats

A total of 142,390 deaths were recorded among the study population between 5th December 2000 and 31st December 2008, during a total of 11.4 million person-years of follow-up. Of these 132,942 (93.4 %) deaths could be linked to a census 2000 record and were included in the analysis. The percentage of linked deaths ranged from 77.4 % among men aged 30–39 years to 95.0 % among men aged 65–94 years, and from 89.1 % among women with other/unknown nationalities to 94.3 % among Swiss women. The percentage linked was also lower in single, widowed and divorced men, and people living in rural communities (Webtable 2).

**Fig. 2:**
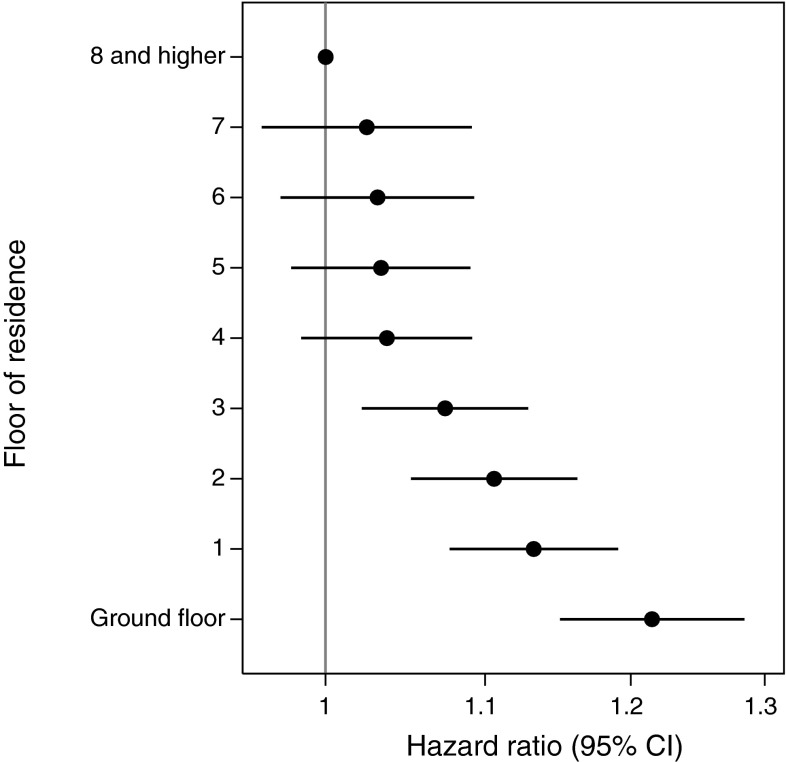
Hazard ratios of death from all causes comparing residents of eighth floor or higher with residents living on lower floors, Switzerland 2001–2008. Hazard ratios were adjusted for age, sex, civil status, nationality, language, religion, education, professional status, type of household and crowding

**Fig. 3:**
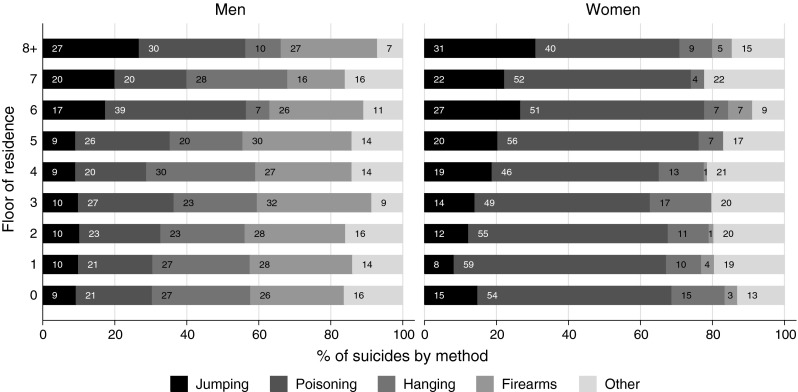
Distribution of method of suicide across floor of residence among 2,697 suicides recorded among 1.5 million residents of buildings with four or more floors, Switzerland 2001–2008

The floor of residence was associated with all-cause and cause-specific mortality. Figure 2 shows fully adjusted HR of death from all causes comparing residents of the eighth floor or higher with residents living on lower floors. Mortality decreased with increasing floor: residents on the ground floor had a 22 % greater hazard of death from any cause compared to residents of the eighth floor and above. The gradient was steepest between ground floor and fourth floor and levelled off from the fourth floor upwards. Table 3 shows the number of deaths included in the analyses and age- and sex-adjusted and fully adjusted HR for all causes and specific causes comparing residents on floors eight and above to ground floors. The association was strongest with transport accidents (fully adjusted HR 2.79; 95 % CI 1.16–6.69), followed by respiratory diseases (fully adjusted HR 1.40; 95 % CI 1.11–1.77), stroke (fully adjusted HR 1.36; 95 % CI 1.07–1.74), cardiovascular diseases (fully adjusted HR 1.35; 95 % CI 1.22–1.49) and lung cancer (fully adjusted HR 1.22; 95 % CI 0.99–1.50).There was little evidence of an association with other causes of death, except for suicide by jumping from a high place. The fully adjusted HR was 0.41 (95 % CI 0.17–0.98) overall (Table 3) and 0.33 (95 % CI 0.11–0.96) in men compared to 0.46 (95 % CI 0.16–1.32) in women (*p* = 0.59 for difference). Figure 3 shows the distribution of methods across floors among the 2,697 suicides recorded during the study period. The percentage of suicides by jumping was higher in women than in men. It increased above the third floor in women and above the fifth floor in men. Among the other means of suicides it was mainly suicide by hanging that became less common on the higher floors.

**Table 3:** Hazard ratios of death from all causes and selected causes comparing residents of ground floor flats to residents of flats on the eight floor or higher, Switzerland 2001–2008

Cause	No. of deaths	Age- and sex-adjusted		Fully adjusted^a^	
		HR	95 % CI	HR	95 % CI
All causes	132,942	1.21	(1.15–1.28)	1.22	(1.15–1.28)
Cardiovascular diseases	47,356	1.37	(1.24–1.51)	1.35	(1.22–1.49)
Myocardial infarction	6,535	1.15	(0.90–1.47)	1.15	(0.90–1.47)
Stroke	7,571	1.37	(1.08–1.74)	1.36	(1.07–1.74)
Respiratory diseases	8,440	1.44	(1.15–1.81)	1.40	(1.11–1.77)
Alcohol related deaths	2,576	0.93	(0.65–1.33)	0.87	(0.59–1.27)
Stomach cancer	1,326	1.30	(0.74–2.29)	1.23	(0.69–2.18)
Lung cancer	7,842	1.25	(1.02–1.53)	1.22	(0.99–1.50)
Breast cancer^b^	3,330	0.89	(0.63–1.26)	0.93	(0.65–1.32)
Prostate cancer^c^	2,773	0.94	(0.59–1.51)	1.01	(0.62–1.63)
Transport accidents^d^	593	2.57	(1.10–6.01)	2.79	(1.16–6.69)
Suicide by any means	2,697	0.71	(0.50–1.00)	0.81	(0.57–1.15)
Suicide by jumping from a high place	336	0.39	(0.17–0.89)	0.41	(0.17–0.98)

Analyses based on 1,500,015 Swiss adults residing in buildings with four and more floors. All models were stratified by building. See main text for ICD-10 codes. *Source*: Swiss National Cohort

*HR* hazard ratio

^a^Adjusted for age, sex, civil status, nationality, language, religion, education, professional status, type of household, household ownership and crowding

^b^Based on 796,043 women

^c^Based on 703,972 men

^d^Including 524 deaths from road traffic accidents (87 %)

There was little evidence for an interaction between floor of residence, socio-economic position of the neighbourhood (measured as quintiles of the Swiss-SEP index) and all-cause mortality (*p* from test of interaction = 0.58). A total of 902,600 individuals (60.2 % of the study population of 1,500,015) had lived at the same address for at least 5 years prior to the beginning of the observation time. The results from the analysis restricted to these individuals were closely similar to the main analysis: the fully adjusted HR comparing all-cause mortality in ground floor residents with those living on floors eight and above was 1.22 (95 % CI 1.13–1.30). Similarly, the corresponding HR was 1.22 (95 % CI 1.15–1.30) during the first 4 years of the observation period, and 1.21 (95 % CI 1.14–1.29) for the second 4-year period.

## Discussion

### Summary of main findings

We examined the association of the floor of residence with all cause and cause specific mortality in the Swiss National Cohort (SNC), a longitudinal study of the entire Swiss population. We included just over 1.5 million people living in buildings with four or more floors and found that mortality from all causes was higher in people living on the ground floor compared to those living on higher floors. An association with floor of residence was evident with causes associated with socio-economically patterned behaviours, such as smoking or diet. For example, mortality from cardiovascular and respiratory diseases declined with higher floors of residence, whereas no association was evident for prostate cancer. Indeed, the pattern of mortality differentials across floors was similar to that found across neighbourhoods of lower and higher socio-economic position [19]. A trend in the opposite direction was observed for suicide by jumping: this method of suicide was more common among residents of higher floors. However, there was no association between suicide by any means and floor of residence.

### Strengths and weaknesses

To our knowledge this is the first large-scale longitudinal study examining the effect of floor of residence on all-cause and cause-specific mortality. The national scope of the study allowed us to include many high-rise buildings of diverse standards that were located in neighbourhoods of different socioeconomic standing. We stratified analyses by building and thus accounted for the socio-economic position of the neighbourhood, level of urbanization, language region and other characteristics of the area. In further analyses we found little evidence that the effect of the floor of residence on mortality was modified by the socio-economic position of the neighbourhood, or that reverse causality might have affected our results. The census collected only limited information about social and economic characteristics of residents, and no information on the health status of residents. Most importantly, the census does not include information on individual or household income, which directly measures material resources [6, 20, 21], or on behavioural and biological risk factors such as smoking, lack of physical activity or blood lipids. We cannot, therefore, exclude with certainty that reverse causality played a role. Switzerland does not have a unique identifier and probabilistic record linkage therefore had to be used to assign mortality to census records. We excluded individuals younger than 30 years at the beginning of the study and persons with missing information on highest achieved education. The highest level of achieved education was used to account for individual socioeconomic position in the analyses. Furthermore, although enumeration in the 2000 census was near-complete [16] we had to exclude 4.9 % individuals with missing information on the floor of residence. Finally, the place of death is not recorded on the death certificate. It therefore remains unclear to what extent suicides by jumping were linked to the respective high-rise buildings.

### Associations with specific causes of death

The association of floor of residence with causes of death such as stroke or lung cancer may be explained by differences in health-related lifestyles and behaviours, for example regarding diet, smoking and levels of physical activity, with healthier lifestyles on higher floors. One contributing factor could be a higher level of physical activity, due to regularly climbing the stairs to the apartment.

The classic study by Morris et al. [22, 23] showed that bus conductors who climbed up and down the stairs of the English double-decker buses had half the coronary mortality of the sedentary drivers. In a recent meta-analysis of cohort studies we found that as little as 20 min of daily moderate to vigorous activity each day was associated with a reduction in all-cause mortality (relative risk 0.86; 95 % CI 0.80–0.92) [24]. The use of elevators in higher buildings will reduce levels of physical activity, which might explain the steeper gradient below five floors (Fig. 2). Environmental exposures may also play a role, for example the higher levels of airborne pollutants, including particulate matter, polycyclic aromatic hydrocarbons or carbon monoxide [25, 26] or higher levels of road traffic noise [27] at lower floor levels. Of note, a previous analysis of the SNC found that aircraft noise was associated with mortality from myocardial infarction, with a dose–response relationship for level and duration of exposure [28].

The increased risk of death from transport accidents in the residents of lower floors is more difficult to explain. A previous study of mortality from road traffic accidents in Switzerland found that the risk of a fatal accident was higher in individuals with low educational attainment, with more pronounced educational differences in pedestrians than in motor vehicle drivers [29]. The risk was also higher among single, widowed and divorced individuals, compared to married persons. In the present study we found no important educational gradient across floors whereas single and widowed individuals tended to live on higher floors. The association between deaths from transport accidents and floor of residence remained when adjusting analyses for these and other variables.

A limitation of our study is the lack of data on individual exposure to road traffic across socio-demographic groups and different transportation modes. Participation in traffic is not assessed in the census and could therefore not be considered in the present analysis. The association with floor of residence might thus simply be due to differences in exposure to traffic, i.e. mileage or time spent in traffic [30]. Some age groups are more exposed to traffic-related risks than others, in particular middle-aged people who are part of the work force and the amount of miles driven decreases with age [31, 32]. The older age of the residents of higher floors supports the notion that differences in exposure to traffic may at least partly explain the association with floor of residence. Furthermore, households with children were more likely to reside on lower floors and these families were perhaps also more likely to be exposed to traffic and its risks. Of note, an earlier analysis of nine European settings, which included the German-speaking part of Switzerland found that the risk of death from transportation injury was higher in men with lower educational level compared with men with higher educational attainment [33].

Suicide by jumping from a high place was substantially increased among residents of the eighth floors and higher compared to those living on the ground floor, which appears to support the claim that “high buildings make people crazy” [12]. The classic study by Fanning [34] of the wives of British and Canadian servicemen who were randomly allocated to floors in three- to four-storey buildings in Germany found that levels of psychological distress in women living on the fourth floor were twice that of women living on the ground floor. Similarly, Gillis [35] reported higher levels of emotional strain among women living on higher floors. A review of studies on housing and mental health found that six out of eight studies showed poorer mental health among residents of higher floor levels [13].

The increased risk of suicide by jumping is of interest to the debate on whether the availability or restriction of access to a method impacts on rates of suicides. Suicide by jumping is the fourth most common method of suicide in Switzerland and only three countries—Germany, Austria and Japan have a similar share of this method [36]. There was little evidence of an increased rate of suicide overall among those living higher up compared to ground floor residents, which argues against the notion that ready access to a method, in this case jumping from a high place of residence, increases the overall rate of suicide. Confidence intervals around the adjusted hazard ratio were, however, wide and we cannot exclude an increase or a reduction in the risk of suicide in those living on floors eight and above. A previous study from Geneva, Switzerland estimated that 62 % of suicides by jumping took place at the home [37]. This method was also found to be more common among women and the elderly [37, 38]. Gunnell and Miller [39] have recently argued that restricting access, for example by placing barriers on bridges, may be less effective than for other methods: jumpers may be less impulsive and less frequently acting in response to same-day crises than people who use more common methods. Restricting access to other methods that are commonly used, highly lethal, and readily accessible in or near the home (such as firearms in the United States or toxic pesticides in developing countries) may be more effective. In the present study 13 % of suicides were by jumping from a high place, compared to 35 % by poisoning and 18 % by use of firearms.

### Generalizability

In many countries the effects of living on different floors in high-rise buildings are likely to be highly contextual and dependent on cultural and socio-economic characteristics of neighbourhoods, levels of crime, traffic, and physical characteristics of buildings. Interestingly, in the present study the association between mortality and floor of residence was not modified by the socio-economic position of the area. The housing situation in Switzerland is fairly unique in comparison to other countries: Switzerland is one of the most affluent nations in the world but has the lowest percentage of home ownership in Europe [40]. Clearly, our results are not applicable to tower blocks in deprived areas of Glasgow, where high-rise housing tends to be in poor physical condition, with poor sound insulation and security [41]. In contrast to the 1960s study of servicemen’s families in Germany [34], residence on higher floors appeared to have a positive effect of on social outcomes and levels of satisfaction among residents of high rise buildings deprived areas of Glasgow. This might be due to an ‘insulating’ effect against social and physical exposures of flats situated on higher floors [41].

### Implications and conclusions

We observed an inverse gradient of all-cause and selected cause specific mortality across floors among residents of high-rise buildings in Switzerland with the exception of suicide by jumping. The pattern of mortality differentials across floors is compatible with the notion that in Switzerland levels of floors are an indicator of socio-economic position, with higher floors reflecting higher socio-economic position, and that the floor of residence is a proxy for social, behavioural and environmental exposures. Floor of residence may thus also act as a confounding factor in studies of mortality or other health outcomes that focus on or include residents of high-rise buildings. Mortality from suicide by jumping was a notable exception to the mortality gradient observed across floors. Further research is needed to better define the characteristics of these suicides.

## Electronic supplementary material

Below is the link to the electronic supplementary material.

Supplementary material 1 (DOC 115 kb)
