# Supplementary material for: High life in the sky? Mortality by floor of residence in Switzerland
Source: Eur J Epidemiol. 2013 May 10;28(6):453–62. doi: 10.1007/s10654-013-9809-8 (PMC3696174; doi:10.1007/s10654-013-9809-8)
Supplement: Supplementary file 1 — Supplementary material 1 (DOC 115 kb) [file 10654_2013_9809_MOESM1_ESM.doc]

**Webtable 1:** Distribution of study population and households across buildings and floors of residence. Swiss National Cohort, Switzerland 2000.

| ***Characteristic*** | ***Population*** | |
| --- | --- | --- |
|  | ***N*** | ***%*** |
|  |  |  |
| **Total** | 1500015 | 100.0 |
|  |  |  |
| **Number of floors in building** | |  |
| 4 | 671210 | 44.7 |
| 5 | 331270 | 22.1 |
| 6 | 179557 | 12.0 |
| 7 | 107834 | 7.2 |
| 8 and above | 210144 | 14.0 |
|  |  |  |
| **Floor of residence** |  |  |
| Ground floor | 218529 | 14.6 |
| 1 | 323716 | 21.6 |
| 2 | 330932 | 22.1 |
| 3 | 281667 | 18.8 |
| 4 | 158605 | 10.6 |
| 5 | 77832 | 5.2 |
| 6 | 42079 | 2.8 |
| 7 | 24409 | 1.6 |
| 8 and above | 42246 | 2.8 |

**Webtable 2:** Percent of deaths between 5^th^ December 2000 and 31^st^ December 2008 within study population linked to 2000 census record. Swiss National Cohort, Switzerland 2000.

|  | ***Men*** | | |  | ***Women*** | | |
| --- | --- | --- | --- | --- | --- | --- | --- |
| ***Characteristic*** | ***Linked deaths*** | | ***Total deaths*** |  | ***Linked deaths*** | | ***Total deaths*** |
|  | ***N*** | ***%*** | ***N*** |  | ***N*** | ***%*** | ***N*** |
| ***Age*** |  |  |  |  |  |  |  |
| 30-39 | 1627 | 77.4% | 2102 |  | 902 | 80.7% | 1118 |
| 40-49 | 3678 | 86.1% | 4273 |  | 2194 | 87.7% | 2501 |
| 50-64 | 13729 | 92.0% | 14928 |  | 8581 | 92.7% | 9258 |
| 65-94 | 45967 | 95.0% | 48403 |  | 56264 | 94.1% | 59807 |
| ***Civil status*** |  |  |  |  |  |  |  |
| Single | 6776 | 89.1% | 7609 |  | 8631 | 93.1% | 9274 |
| Married | 40387 | 96.5% | 41863 |  | 16165 | 96.5% | 16754 |
| Widowed | 11286 | 88.5% | 12759 |  | 34989 | 92.5% | 37834 |
| Divorced | 6552 | 87.7% | 7475 |  | 8156 | 92.5% | 8822 |
| ***Nationality*** |  |  |  |  |  |  |  |
| Switzerland | 51721 | 94.0% | 55019 |  | 53180 | 94.3% | 56397 |
| Rest of Europe | 11878 | 90.5% | 13121 |  | 13248 | 90.8% | 14589 |
| Other / unknown | 1402 | 89.5% | 1566 |  | 1513 | 89.1% | 1698 |
| ***Language region*** |  |  |  |  |  |  |  |
| German | 42291 | 94.2% | 44885 |  | 44287 | 94.8% | 46719 |
| French | 20214 | 91.4% | 22114 |  | 21061 | 91.4% | 23053 |
| Italian | 2493 | 92.3% | 2701 |  | 2591 | 89.2% | 2905 |
| Missing | 3 | 50.0% | 6 |  | 2 | 28.6% | 7 |
| ***Urbanization*** |  |  |  |  |  |  |  |
| Urban | 34849 | 94.9% | 36726 |  | 38481 | 95.3% | 40365 |
| Peri-urban | 22933 | 92.4% | 24826 |  | 22206 | 91.8% | 24193 |
| Rural | 7216 | 88.6% | 8148 |  | 7252 | 89.3% | 8119 |
| Missing | 3 | 50.0% | 6 |  | 2 | 28.6% | 7 |
| ***Total*** | 65001 | 93.3% | 69706 |  | 67941 | 93.5% | 72684 |

**Webtable 3:** Age, sex and fully adjusted hazard ratios of death from all causes and selected causes among the 1’500’515 Swiss adults (older than 30 years at baseline) residing in buildings with four and more floors; reference category - residents of the flats on the eight floor or higher; All models stratified by building; Switzerland 2001-2008. Source: Swiss National Cohort.

|  | Adjusted for age and sex | | Fully adjusted ^a^ | |
| --- | --- | --- | --- | --- |
|  | HR | 95% CI | HR | 95% CI |
| ***Floor of residence*** Ground floor | 1.21 | (1.15, 1.28) | 1.22 | (1.15, 1.28) |
| 1 | 1.13 | (1.08, 1.19) | 1.13 | (1.08, 1.19) |
| 2 | 1.11 | (1.05, 1.16) | 1.11 | (1.05, 1.16) |
| 3 | 1.07 | (1.02, 1.13) | 1.07 | (1.02, 1.13) |
| 4 | 1.04 | (0.99, 1.10) | 1.04 | (0.99, 1.09) |
| 5 | 1.04 | (0.98, 1.10) | 1.03 | (0.98, 1.09) |
| 6 | 1.03 | (0.97, 1.09) | 1.03 | (0.97, 1.09) |
| 7 | 1.02 | (0.96, 1.09) | 1.02 | (0.96, 1.09) |
| 8 and above | 1.00 |  | 1.00 |  |
| ***Gender*** Male | 1.00 |  | 1.00 |  |
| Female | 0.55 | (0.54, 0.56) | 0.48 | (0.47, 0.48) |
| ***Nationality***  Switzerland | 1.00 |  | 1.00 |  |
| Rest of Europe | 0.76 | (0.74, 0.78) | 0.84 | (0.81, 0.87) |
| Other / unknown | 0.55 | (0.50, 0.61) | 0.67 | (0.60, 0.75) |
| ***Civil status*** Single | 1.43 | (1.39, 1.47) | 1.44 | (1.38, 1.50) |
| Married | 1.00 |  | 1.00 |  |
| Widowed | 1.31 | (1.27, 1.34) | 1.34 | (1.29, 1.39) |
| Divorced | 1.39 | (1.35, 1.43) | 1.39 | (1.34, 1.45) |
| ***Religion*** Protestant | 1.00 |  | 1.00 |  |
| Catholic | 0.94 | (0.92, 0.96) | 0.98 | (0.96, 1.00) |
| No affiliation | 0.97 | (0.94, 1.00) | 0.98 | (0.95, 1.02) |
| Other / unknown | 0.95 | (0.92, 0.98) | 1.02 | (0.98, 1.05) |
| ***Language*** German | 1.00 |  | 1.00 |  |
| French | 0.99 | (0.95, 1.02) | 0.99 | (0.95, 1.03) |
| Italian | 0.80 | (0.77, 0.84) | 0.91 | (0.86, 0.95) |
| Other | 0.65 | (0.62, 0.68) | 0.77 | (0.73, 0.81) |
| ***Education*** Compulsory or less | 1.03 | (1.01, 1.05) | 1.05 | (1.03, 1.07) |
| Upper secondary | 1.00 |  | 1.00 |  |
| Tertiary | 0.86 | (0.84, 0.88) | 0.91 | (0.88, 0.93) |
| ***Professional status*** Top management and independent professions | 0.90 | (0.81, 1.01) | 0.96 | (0.86, 1.08) |
| Other self-employed | 1.10 | (1.03, 1.18) | 1.12 | (1.05, 1.20) |
| Professionals and senior management | 0.79 | (0.73, 0.86) | 0.84 | (0.77, 0.91) |
| Supervisors/low level management and skilled labour | 1.00 |  | 1.00 |  |
| Unskilled employees and workers | 0.98 | (0.93, 1.05) | 1.05 | (0.98, 1.11) |
| In paid employment, not classified elsewhere | 1.38 | (1.31, 1.44) | 1.42 | (1.36, 1.49) |
| Unemployed/job-seeking | 1.82 | (1.69, 1.96) | 1.87 | (1.74, 2.02) |
| Not in paid employment | 1.97 | (1.89, 2.05) | 2.03 | (1.95, 2.11) |
| ***Type of household*** Single person household | 1.00 |  | 1.00 |  |
| Couple without children | 0.81 | (0.79, 0.83) | 1.04 | (1.00, 1.08) |
| Household with children | 0.62 | (0.60, 0.65) | 0.87 | (0.82, 0.92) |
| Single parent household | 0.84 | (0.79, 0.91) | 0.91 | (0.84, 0.98) |
| Other | 0.97 | (0.92, 1.03) | 0.97 | (0.91, 1.03) |
| ***Household ownership*** Rented flat | 1.00 |  | 1.00 |  |
| Owned flat | 0.84 | (0.77, 0.91) | 0.87 | (0.79, 0.94) |
| Other | 0.85 | (0.77, 0.95) | 0.90 | (0.81, 1.00) |
| ***Household crowding*** (per additional person/room) | 0.82 | (0.79, 0.85) | 1.03 | (0.99, 1.07) |

^a^ Adjusted for age, sex, civil status, nationality, language, religion, education, professional status, type of household, household ownership and crowding
